# Psychiatric Disorders Among Older Black Americans: Within- and Between-Group Differences

**DOI:** 10.1093/geroni/igaa007

**Published:** 2020-04-15

**Authors:** Robert Joseph Taylor, Linda M Chatters

**Affiliations:** 1 School of Social Work, University of Michigan, Ann Arbor; 2 Institute for Social Research, University of Michigan, Ann Arbor; 3 Department of Health Behavior & Health Education, School of Public Health, University of Michigan, Ann Arbor

**Keywords:** African American, Anxiety, Depression, Mental health, Race, Serious mental illness

## Abstract

Psychiatric disorders impose significant personal, social, and financial costs for individuals, families, and the nation. Despite a large amount of research and several journals focused on psychiatric conditions, there is a paucity of research on psychiatric disorders among Black Americans (i.e., African Americans and Black Caribbeans), particularly older Black Americans. The present literature review examines research on psychiatric disorders among older Black Americans and provides a broad overview of research findings that are based on nationally representative studies. Collectively, this research finds: (1) older African Americans have lower rates of psychiatric disorders than younger African Americans; (2) family support is not protective of psychiatric disorders, whereas negative interaction with family members is a risk factor; (3) everyday discrimination is a risk factor for psychiatric disorders; (4) both older African Americans and African American across the adult age range have lower prevalence rates of psychiatric disorders than non-Latino whites; (5) Black Caribbean men have particularly high rates of depression, posttraumatic stress disorder, and suicide attempts; and (6) a significant proportion of African American older adults with mental health disorders do not receive professional help. This literature review also discusses the “Race Paradox” in mental health, the Environmental Affordances Model, and the importance of investigating ethnicity differences among Black Americans. Future research directions address issues that are directly relevant to the Black American population and include the following: (1) understanding the impact of mass incarceration on the psychiatric disorders of prisoners’ family members, (2) assessing the impact of immigration from African countries for ethnic diversity within the Black American population, (3) examining the impact of racial identity and racial socialization as potential protective factors for psychiatric morbidities, and (4) assessing racial diversity in life-course events and their impact on mental health.

**Translational Significance**

There is a paucity of research on psychiatric disorders and serious mental health among Black Americans (i.e., African Americans and Black Caribbeans) in general and especially among older black adults. Research generally finds that despite lower socioeconomic status and more physical health problems, older African Americans have lower rates of psychiatric disorders than older non-Latino whites. Among those who have psychiatric disorders, older Black Americans are significantly less likely to use mental health services than older non-Latino whites.

The 75th Anniversary of The Gerontological Society of America gives us the opportunity to reflect on the organization’s contributions in advancing innovations in theory, research, and practice in service to older adults. Projected increases in both the overall size and the racial/ethnic composition of the U.S. older population underscore the urgency of addressing limitations in our knowledge concerning mental health issues for older adults, and especially elders from racial and ethnic minority groups. In the spirit of “Honor the Past, Enrich the Future,” this article reviews the literature on psychiatric disorders among older Black Americans and discusses substantive foci and strategies for enhancing knowledge in this area. It is hoped that this article builds on continuing efforts to understand and support the mental well-being of diverse groups of older persons.

Psychiatric disorders are extremely debilitating and represent a major public health issue. The estimated economic cost of major depressive disorder (MDD) alone in the United States was $210.5 billion in 2010 (Greenberg, Fournier, Sisitsky, Pike, & Kessler, 2015). Roughly half of those costs were for workplace issues such as missed days at work and reduced productivity while at work. The other half were due to direct costs associated with medical services, prescription drugs for MDD, and comorbid psychiatric disorders, as well as costs related to suicide (Greenberg et al., 2015). Having a psychiatric disorder, particularly one with early onset, is associated with serious negative outcomes over the life course (Kessler, Bromet, de Jonge, Shahly, & Wilcox, 2017). For instance, individuals with early-onset psychiatric disorders have a higher likelihood of lower levels of education and occupational attainment, reduced incomes over the life course, higher rates of unemployment, and higher rates of marital instability (Kessler et al., 2017). Despite an extensive literature on psychiatric disorders for non-Latino whites, research on African Americans is extremely limited, especially in regards to older African Americans.

This review of the literature on psychiatric disorders among older African Americans examines both within group differences (e.g., sociodemographic) among African Americans, as well as comparative research with non-Hispanic whites. We also include available research on older Black Caribbeans in the United States. Separate literature reviews that specifically explore psychiatric disorders among older Asian Americans or older Latinos are forthcoming in this journal. Consequently, our review of the literature does not cover research on the mental health of these older populations. We review studies that specifically focus on diagnosed psychiatric disorders rather than symptoms of depression (Center for Epidemiologic Studies Depression scale [CES-D]) or symptoms of anxiety (Patient Health Questionnaire) and research that is based on national data. Finally, definitions of who is an older adult vary significantly. Most research in this area defines older adults as being 55 years of age and older (Ford et al., 2007), some research uses 60 years and above (Jimenez, Alegría, Chen, Chan, & Laderman, 2010), and other work defines older adults as persons 50 years and above (Woodward, Taylor, Abelson, & Matusko, 2013).

Note that in reviewing the literature our use of the term African American(s) relates to persons of African descent who are native born to the United States, while Black Caribbean(s) are individuals who have ties and trace their ancestry to the Caribbean region (i.e., immigrants). The overall term Black American(s) indicates both African Americans and Black Caribbeans. The literature review begins with a discussion of diversity within the Black American population, followed by information concerning the Collaborative Psychiatric Epidemiology Studies (CPES), psychiatric disorders across the life course for African Americans, psychiatric disorders among Black Americans (with a focus on gender and ethnicity/nativity differences). Following this, we present research specifically on psychiatric disorders among older African Americans including protective factors that are associated with lower likelihood of psychiatric disorders, and research on racial discrimination and microaggressions. The final two substantive sections consider race and prevalence of psychiatric disorders among older adults and information on use of services for psychiatric disorders among older African Americans. The literature review closes with a section focused on future research directions and conclusions.

## Diversity Within the Black American Population

Within the context of the United States, black race remains a problematic construct that portrays Black Americans as a monolithic and undifferentiated group. Racial designations have long-standing predominance in the United States, such that considerations of ethnicity, immigration, and other differences within the Black American population in the United States are only slowly informing discussions of variability within this population group. In practice, the use of an undifferentiated racial category obscures the presence of Caribbean blacks as a distinct ethnic subgroup within the larger Black American population. Consequently, despite their historical presence and growth as a segment of the population (Anderson & Lopez, 2018), blacks within the United States who have origins and/or connections to the Caribbean region remain largely invisible. For example, black immigrants from Caribbean countries (e.g., Jamaica and Haiti) represent sizable proportions of the black populations in major metropolitan and neighboring areas in New York/New Jersey, Boston, Miami and West Palm Beach/Boca Raton and Fort Lauderdale (Logan & Deane, 2003, Table 2). In fact, among foreign-born blacks in the United States, in 2016, close to half were from the Caribbean region (Anderson & Lopez, 2018).

In using the broad category of Black Americans, social and health scientists have also ignored the presence of Caribbean blacks within the black racial category. This practice and omission is problematic as it obscures important differences in national heritage, cultural practices, demographic profiles, and life experiences that distinguish native-born African Americans and Black Caribbeans (Logan & Deane, 2003). Further, these differences are potentially relevant for health and social phenomena (e.g., health beliefs and practices). The ability to differentiate Black Caribbeans and African Americans is particularly important with regards to psychiatric disorders because of important differences in prevalence rates and the patterns and distribution of disorders for these two populations (Williams et al., 2007).

## Collaborative Psychiatric Epidemiology Studies

Studies of psychiatric disorders among racial and ethnic minority populations are exceedingly rare, especially studies based on national probability samples. This is due to several factors. First, historically, there has been a lack of racial and ethnic minority researchers who investigate these issues. Second, the low prevalence rate for some psychiatric disorders has meant that surveys with large numbers of respondents are needed to have enough power to adequately conduct analysis. Even surveys with large numbers of African American respondents (say 1,000) will have insufficient cases to adequately conduct analysis of psychiatric disorders for African Americans aged 55 years and older. Third, measuring psychiatric disorders within a survey requires complicated, detailed, and time-consuming survey protocols. In contrast, assessing depressive or anxiety symptoms has been standardized in available measures (e.g., CES-D) requiring relatively few items. Fourth, African Americans are more likely to reside in large urban cities where it tends to be more expensive to conduct surveys.

The advent of the CPES addressed these issues and contains data about the prevalence of mental disorders, their associated impairments, and their treatment. The Collaborative Psychiatric Epidemiology Surveys is comprised of three linked data sets: the National Comorbidity Survey—Replication (NCS-R), the National Latino and Asian American Study (NLAAS), and the National Survey of American Life: Coping with Stress in the 21st Century (NSAL). All three studies were collected by the Survey Research Center at the University of Michigan (Heeringa et al., 2004; Pennell et al., 2004). The CPES surveys share the national multistage area probability sample designs, share a common set of objectives and instrumentation and were designed to allow integration of design-based analysis weights to combine data sets as though they were a single, nationally representative study (Heeringa et al., 2004). At the same time, however, each survey has unique features in their national area probability samples that complement one another. The NCS-R is designed to be representative of the U.S. population in general and includes face-to-face interviews with 9,282 adults. The NSAL was designed to be representative of blacks in the United States and is based upon a national household probability sample of 6,082 African Americans, non-Latino whites, and blacks of Caribbean descent (Jackson, Knight, & Rafferty, 2004). The NLAAS was designed to be representative of Latinos and Asian Americans in the United States (see Heeringa et al., 2004 for a more detailed discussion of the CPES samples). The CPES surveys used the Diagnostic and Statistical Manual of Mental Disorders, Fourth Edition (DSM-IV) World Mental Health Composite International Diagnostic Interview, a fully structured diagnostic interview used to assess mental disorders (Kessler & Üstün, 2004).

Collectively, the CPES has led to ground-breaking studies of the prevalence and correlates of psychiatric disorders among African American, Asian American, Latinx, and white populations. Although the numbers of racial and ethnic minority older respondents aged 55 years and older in the CPES are relatively small for prevalence studies, these numbers are larger than what has been historically available. Many of the studies reviewed here use data from either the NSAL or from all three of the CPES data sets.

Despite the many advantages of the CPES studies, there are some important limitations. Prevalence rates tend to be lower for older adults than for adults 18–54. Consequently, much of the analysis presented here is for the general categories of mood disorders or anxiety disorders. There are not enough cases to examine more discrete disorders such as social phobia, posttraumatic stress disorder (PTSD) and obsessive-compulsive disorder (OCD). Due to small sample sizes, there are instances where only symptoms (e.g., CES-D) can be analyzed as opposed to disorders. This is especially true of analyses that examine risk and protective factors for mental health problems as opposed to prevalence rates for disorders. The major limitation of the CPES is the age of the data sets; they are now over 15 years old. Nonetheless, they remain the only data sets with enough cases of racial and ethnic minority older adults to conduct meaningful analysis on psychiatric disorders. Prior to the advent of the NSAL and NLAAS data sets, reliable, nationally representative information on psychiatric disorders for these groups was either scarce or nonexistent. Finally, the NSAL and NLAAS contain survey items that are particularly relevant to minority populations and researchers including questions on topics such as racial identity, church-based social support, racial socialization, acculturation and more extensive items on religious participation, and family/friendship support. Considering the historic scarcity of national probability-based data sets on minority mental health, the CPES data sets remain the best and most comprehensive data for these issues.

## Psychiatric Disorders Across the Life Course for African Americans: Age and Race Differences

A small but growing body of research on psychiatric disorders among African Americans indicates that older African Americans have lower prevalence of mood and anxiety disorders than their younger counterparts (it is important to note that 12-month prevalence indicates the proportion of the population that experienced a psychiatric disorder over the previous 12 months; lifetime prevalence, the proportion of the population that experienced a psychiatric disorder at some point in life). For instance, older African Americans have lower lifetime prevalence rate of MDD (Williams et al., 2007) and are also less likely than those aged 18–29 to have: (1) complicated depression versus affective depression, and (2) physical versus affective depression (Scott, Matsuyama, & Mezuk, 2011). Affective depression refers to depression that is not attributed to health problems and complicated depression refers to depression that is sometimes attributed to physical problems, while physical depression is always attributed to physical health problems (Scott et al., 2011, p. 67). Older African Americans had lower prevalence rates of 12-month generalized anxiety disorder, social anxiety (or social phobia), PTSD, and agoraphobia without panic (Himle, Baser, Taylor, Campbell, & Jackson, 2009). African Americans aged 55 years and older were less likely to have 12-month and lifetime panic disorder (Levine et al., 2013) and 12-month and lifetime OCD than their younger counterparts (Himle et al., 2008). Lastly, Joe, Baser, Breeden, Neighbors, and Jackson (2006) conducted the most definitive study of the prevalence of suicidal behaviors among African Americans to date. They found that for African Americans and Black Caribbeans combined, older respondents had the lowest rates of suicidal attempts and ideation. There are also separate analyses of psychiatric disorders among African American men (Mays et al., 2018) and mothers (Boyd, Joe, Michalopoulos, Davis, & Jackson, 2011).

There are two possible explanations for the lower prevalence rates among older African Americans. First, lower prevalence rates for psychiatric disorders could be due to specific life experiences that characterized this group of older adults (i.e., cohort effect). Likewise, the higher prevalence rates among younger African American adults may reflect the experiences of their particular age cohort. If this is the case, one would expect that as younger and middle-aged African American adults move into older age, they will report higher prevalence rates for psychiatric disorders. Second, age differences could also be due to a selection effect, that is, individuals who have serious mental health problems have higher rates of mortality. This is especially true of those who experience early onset and have a longer duration of psychiatric disorders. Finally, because these are self-report measures, older adults who had mental health issues at a younger age may not recall them or decline to report them due to their stigmatized nature.

Turning to findings for race differences, prevalence rates of psychiatric disorders have been very consistent in indicating that African Americans have lower prevalence rates for most 12-month and lifetime psychiatric disorders than non-Latino whites. This includes lower rates of lifetime MDD (Williams et al., 2007), 12-month general anxiety disorder, social anxiety (or social phobia), and agoraphobia without panic (Himle et al., 2009). African Americans were also less likely than non-Latino whites to have 12-month or lifetime panic disorder (Levine et al., 2013). Although no racial differences were found for 12-month MDD (Williams et al., 2007), African Americans had higher persistence of MDD than non-Latino whites, indicating that for African Americans MDD is more chronic in nature (Williams et al., 2007). African Americans and non-Latino whites were comparable in terms of the persistence of anxiety or substance use disorders (Vilsaint et al., 2019), as well as in the prevalence of 12-month PTSD (Himle et al., 2009), However, African Americans were more likely than non-Latino whites to develop PTSD following a traumatic event (McLaughlin et al., 2019). The average age of onset of MDD for African Americans was 25.9 years for 12 months and 26.7 years for lifetime which is roughly the same for non-Latino whites and other populations (Gonzalez et al., 2010; González et al., 2010b). In addition, among non-Latino whites, obesity was associated with 12-month major depressive episode, panic disorder, and PTSD, but obesity was not associated with disorders among African Americans and Black Caribbeans (Rosen-Reynoso et al., 2011: Table 2).

Research based on several national samples and using various measures of psychopathology consistently finds that overall African Americans have lower prevalence rates than non-Latino whites for most mood and anxiety disorders (Mezuk et al., 2013). This is despite the fact that African Americans have greater risk factors for psychiatric disorders including higher levels of poverty, poorer health, higher rates of discrimination, and greater likelihood of residing in neighborhoods with higher levels of social stressors (e.g., crime). This constellation of risk factors for psychiatric disorders would predict that African Americans would have higher levels of psychiatric disorders. Researchers have termed this phenomenon the “race paradox” in mental health. Research has explored several factors and mechanisms in an attempt to explain this race paradox. Studies have focused on the role of family support networks (Mouzon, 2013), friendship networks (Mouzon, 2014), and religion (Mouzon, 2017), while others examine gender differences (Erving, Thomas, & Frazier, 2019), gender and age differences (Barnes, Keyes, & Bates, 2013), as well as other issues (Barnes & Bates, 2017). However, to date, none of these studies has adequately explained the underlying reasons for this paradox.

The Environmental Affordances Model developed by Jackson and colleagues (Jackson et al., 2010) is a promising theory that may explain this race paradox in mental health. Jackson and colleagues (Jackson et al., 2010; Mezuk et al., 2010; Mezuk et al., 2013) argue that mental and physical health disorders are functionally related. In particular, lower rates of psychiatric disorders of African Americans may come at the expense of higher rates of physical health problems. The Environmental Affordances Model states that African Americans cope with stress by engaging in unhealthy behaviors (e.g., smoking, alcohol use, eating of comfort foods, and overeating) as a means to cope with stressors. Unhealthy behaviors may reduce the likelihood of developing a psychiatric disorder, but increase the likelihood of developing chronic physical health problems in later life (e.g., diabetes, heart disease). Ongoing research by this group provides empirical evidence to support the Environmental Affordances Model (Jackson et al., 2010; Mezuk et al., 2010; Mezuk et al., 2013), but more work is needed.

## Psychiatric Disorders Among Black Americans: Gender and Ethnicity/Nativity Differences

One of the more intriguing sets of findings concern gender differences in psychiatric disorders among Black Caribbeans and African Americans. Studies using the NSAL data indicate that for both adults (Williams et al., 2007) and older adults (Woodward et al., 2013), Black Caribbean men have higher rates of MDD than African American men. Further, the rate of major depression for Black Caribbean men is similar to that for Black Caribbean women and African American women (Williams et al., 2007). Black Caribbean men also had higher rates of suicide attempts than Black Caribbean women, African American women, and African American men (Joe et al., 2006). These findings are significant given prior research indicating that women typically have higher rates of depression and suicide attempts compared to men. Black Caribbean men also had higher rates of PTSD than Black Caribbean women, as well as the highest rates for PTSD of all of the six gender and racial groups studied (African American, Non-Latino white, Black Caribbean; Himle et al., 2009).

Mays and associates (Mays et al., 2018) conducted a detailed analysis of ethnicity differences in psychiatric disorders for African American men, Black Caribbean men born in the United States and foreign-born Black Caribbean men. They found that in comparison to African American men, U.S.-born Black Caribbean men had higher prevalence of MDD, PTSD, panic disorder, and alcohol and drug use disorders. Further, U.S.-born Black Caribbean men had more chronic generalized anxiety disorder, panic disorder, and substance use disorders than the other groups of black men. These findings in conjunction with previous research indicate that U.S.-born Black Caribbean men are particularly vulnerable to mental health problems and that both ethnic and nativity differences are important to examine when conducting analysis of the black population.

## Psychiatric Disorders Among Older African Americans

Although relatively small, several early studies of note explore the mental health status of older African Americans. Baqar Husaini from Tennessee State University conducted numerous studies on mental health of older African Americans in Nashville, Tennessee (Husaini et al., 1991). In addition, several Epidemiological Catchment Area (ECA) studies examined age differences in psychiatric disorders among African Americans (Somervell, Leaf, Weissman, Blazer, & Bruce, 1989). Recent literature reviews on race and mental health among older adults are available including a review of research on racial and ethnic minorities (Trinh et al., 2019) and one on depression among older African Americans (Pickett, Bazelais, & Bruce, 2013). Lastly, many of the articles cited in this review are published in psychiatric journals, most notably the *American Journal of Geriatric Psychiatry* and the *International Journal of Geriatric Psychiatry*. These are journals that may not be familiar to social scientists and nonmedical (e.g., geriatric social workers, psychologists) practitioners.

Researchers have long been interested in how the general public defines and understands mental illness and psychiatric disorders. In a small qualitative study of older African Americans, Akinyemi and colleagues (2018) found that language used by older African Americans to describe depression is largely consistent with DSM-IV criteria. However, in describing depression, older African Americans also used terms such as loneliness, helplessness, and social isolation, which do not align with DSM-IV criteria for depression. Nonetheless, it is important to note that loneliness and social isolation are strong correlates of depression and other psychiatric disorders among both older African Americans and whites (Taylor, Taylor, Nguyen, & Chatters, 2018).

Participants in a large randomized trial for older adults with mental health and substance use issues provided information about how cultural beliefs shape understanding the causes of mental health problems. Older African Americans indicated that some of the major causes of mental illness were stress, loss of a family member or friend, and financial issues (Jimenez, Bartels, Cardenas, Dhaliwal, & Alegría, 2012). Roughly one-third of African Americans and whites believed that medical illness was a cause of mental illness, while Asian Americans and Latinos were less likely to endorse this belief.

Several studies of psychiatric disorders among older African Americans are based on the National Survey of American Life. Using the NSAL, Ford and colleagues (2007) established the first ever national estimates of psychiatric disorders among older African Americans, indicating that 23% of older (55+) African Americans reported at least one lifetime disorder and 8.54% reported at least one 12-month disorder. The most prevalent 12-month disorders were PTSD, major depression, and social phobia, while the most prevalent lifetime disorders were alcohol abuse, PTSD, and major depression. Age, sex, education, and region were significantly associated with odds of having a lifetime disorder. One of the more noteworthy findings was regional differences indicating that older African Americans who resided in the South were less likely to have any lifetime anxiety disorder, any lifetime substance disorder, and overall any lifetime disorder than their counterparts outside of the South. The lower rates of lifetime disorders were thought to be attributable to higher levels of religious participation and more supportive family networks for older African Americans in the South as compared to other regions (Ford et al., 2007).

## Protective Factors and Psychiatric Disorders

Two studies investigated potential protective factors for psychiatric disorders among older African Americans. A study of religious involvement and DSM-IV disorders (Chatters et al., 2008) found that the frequency of religious service attendance was protective against mood disorders, but not anxiety disorders. Controlling for physical health conditions and disability (i.e., mobility and self-care), frequency of service attendance was inversely associated with the odds of having both lifetime and 12-month mood disorder. Frequency of service attendance and subjective religiosity were also inversely associated with having any lifetime disorder, but these relationships were not maintained when controlling for other religiosity variables (Chatters et al., 2008).

Lincoln and colleagues’ (2010) study of family relationships and psychiatric disorders found that emotional support from family members was unrelated to having a lifetime mood or anxiety disorder and unrelated to the number of lifetime mood and anxiety disorders. Negative interaction with family members, however, was significantly and positively associated with having a lifetime mood disorder, a lifetime anxiety disorder and the number of lifetime mood and anxiety disorders. Collectively, these findings indicate that emotional support from family is not protective for psychiatric disorders, but negative interaction with family members (i.e., criticisms, arguments) is a potent risk factor for or consequence of poor mental health among older African Americans. Given the cross-sectional nature of the data, causal relationships between negative interactions with family members and anxiety and mood disorders could not be established. That is, to what extent do negative interactions with family precipitate the development of anxiety or mood disorders, versus negative family interactions are an outcome of having a disorder.

In a novel study of psychiatric disorders, Peterson, Chatters, Taylor, and Nguyen (2014) examined the impact of 12-month mood disorders, 12-month anxiety disorders, and lifetime suicidal ideation on assessments of happiness and life satisfaction among a sample of older African Americans who reported at least one psychiatric disorder. Study findings indicated that having a 12-month mood disorder was negatively associated with both happiness and their satisfaction. Given that the symptoms of mood disorders include depressed mood, loss of interest or pleasure, and feelings of worthlessness, its association with lower levels of happiness and life satisfaction is not surprising. Anxiety disorders and suicidal ideation, however, were unrelated to happiness, indicating that even among older African Americans with diagnosed psychiatric disorders, having a 12-month anxiety disorder or previous suicidal ideation does not mean that a person will be unhappy. Arguably the major finding from this study was that having a lifetime DSM-IV psychiatric disorder is not necessarily associated with dissatisfaction with life and unhappiness. Within this group of older African Americans with a diagnosed psychiatric disorder, close to a third of respondents reported that they were very satisfied with their lives, while an equal portion indicated that they were very happy overall.

Similar to the state of research on psychiatric disorders and mental health in general, suicide, and suicidal behaviors among older African Americans and Black Caribbeans is severely understudied. Using NSAL data, Joe, Ford, Taylor, and Chatters (2014) found that the estimated lifetime prevalence of suicidal ideation and attempts among older African Americans and Black Caribbeans was 6.1% and 2.1%, respectively. No significant ethnic differences for prevalence rates for suicide attempts or ideation were found. Men were more likely than women to have had suicidal ideations and ideation was positively associated with several psychiatric disorders (MDD, dysthymia, panic disorder, agoraphobia, generalized panic disorder, PTSD, alcohol abuse, and binge eating). Older black adults with multiple psychiatric disorders were more likely to be at risk for suicidal attempts and ideations (Joe et al., 2014). Lastly, roughly one in four attempters and two in five ideators never sought treatment for their emotional or psychological problems.

## Discrimination, Microaggressions, and Psychiatric Disorders

The last 25 years has seen a tremendous growth in research on the importance of discrimination as a major stressor that negatively impacts mental and physical health. Two types of discrimination are typically examined. Major or acute discrimination includes being unjustly fired, not being able to rent an apartment, not being hired. Although it has been suggested that these experiences may be justified and are not reflections of discriminatory treatment, numerous audit studies (e.g., apartment rentals, job applications) have proven high rates of discrimination in a range of areas (Williams & Mohammad, 2013). For example, Pager’s (2003) experimental audit study involving matched pairs of white and African American male testers applying for real jobs found that black men without a criminal record were significantly less likely to receive a call back than white men with a criminal record. She further noted that employers seemed to expect that African American job applicants had a criminal background (Pager, 2003).

Everyday discrimination, on the other hand, comprises types of unfair treatment that are more chronic in nature including being treated with less courtesy, treated with less respect, receiving poor restaurant service, being perceived as not smart, being perceived as dishonest, or being feared, insulted, and harassed and is assessed by the Everyday Discrimination Scale (Essed, 1991; Williams & Mohammad, 2013). Several reviews of the literature document the deleterious effects of everyday discrimination on physical and mental health (Lewis, Cogburn, & Williams, 2015; Pascoe & Smart Richman, 2009; Williams & Mohammed, 2013). For instance, in review of this literature, Lewis and colleagues (2015) note that discrimination negatively impacts a variety of objective physical health outcomes including breast cancer, hypertension, and all-cause mortality.

A final class of discriminatory treatment is the concept of microaggressions. Racial microaggressions have been defined as “brief and commonplace daily verbal, behavioral, or environmental indignities, whether intentional or unintentional, that communicate hostile, derogatory, or negative racial slights and insults toward people of color” (Sue et al., 2007). Microaggressions are similar to several items of the Everyday Discrimination Scale, such as being treated with less courtesy, treated with less respect, and being perceived as not smart. While the study of microaggressions is prevalent in the psychological literature (see Wong, Derthick, David, Saw, & Okazaki, 2014 for an exhaustive review), everyday discrimination is more commonly researched in the sociological and public health literatures. Overall, research indicates that African Americans have higher levels of everyday discrimination than whites and are more likely to attribute discrimination to racial causes rather than nonracial causes (Kessler, Mickelson, & Williams, 1999).

Relative to the large body of research on the impact of discrimination, very little research examines this issue among older African Americans (Mouzon, Taylor, Nguyen, Ifatunji, & Chatters, in press). Two recent articles investigated the impact of discrimination on psychiatric disorders among older African Americans. Mouzon, Taylor, Keith, Nicklett, and Chatters (2017), conducting one of the first analysis of this issue, found that both racial and nonracial discrimination was associated with worse mental health. Older African Americans who experienced higher levels of overall everyday discrimination had higher odds of any psychiatric disorder, any lifetime mood disorder, any lifetime anxiety disorder, and more lifetime DSM-IV disorders, as well as more depressive symptoms and serious psychological distress. Associations between discrimination and worse mental health were found for discrimination that was attributed to both racial and nonracial reasons (e.g., gender, weight status). Overall, out of 18 possible relationships between discrimination and mental health outcomes, 16 were significant. In a related article, Kim and colleagues’ (2017) investigation of racial discrimination among older African Americans and Black Caribbeans found that discrimination was significantly associated with increased odds of having a 12-month psychiatric disorder. Further, regional differences indicated that this relationship was stronger for older blacks in the West as compared to those residing in the South. Noted areas for future investigation include examining how different regional patterns for older African Americans (primarily South) and Black Caribbeans (primarily Northeast and Florida) impact experiencing everyday discrimination and whether the two groups experience different levels and types of racial discrimination. For example, do Black Caribbean older adults attribute everyday discrimination occurrences to immigrant status?

## Race and Prevalence of Psychiatric Disorders Among Older Adults

As one of the first major studies of racial differences in psychiatric disorders among older adults, Jimenez and colleagues (2010) used the CPES to investigate racial and nativity differences in 12-month and lifetime psychiatric disorders among adults who were 60 years of age and older. They found that African Americans were less likely than non-Latino whites to have a lifetime major depressive episode and Black Caribbeans were less likely than non-Latino whites to have lifetime general anxiety disorder and social phobia. No significant differences between foreign and native-born Black Caribbeans were found for 12-month and lifetime prevalence rates, but this could be due to the small number of Black Caribbeans in the analysis.

Woodward and colleagues (2012) followed up on this work by also using the CPES data and expanding the analysis in several ways, including investigating demographic differences, analysis of both non-Latino whites and African Americans as the reference/comparison group, and expanding the age range of the analysis to persons aged 55 years and older. Non-Latino whites, Black Caribbeans and Latinos were all more likely than African Americans to have a lifetime mood disorder. Asians and non-Latino whites were more likely to have an anxiety disorder. Analysis among African Americans showed that having lower family incomes was associated with a higher prevalence of both mood and anxiety disorders.

Innovative research by Kim and colleagues (2011) examined the relationship between self-reported mental health and psychiatric disorders and found that persons with poor self-rated mental health were more likely to report both 12-month mood and anxiety disorders. However, this relationship was much stronger for non-Latino whites than for African Americans, Asians, and Latinx older adults. Other analysis (Aranda et al., 2012) found that African Americans were less likely than non-Latino whites to have lifetime major depression and that Black Caribbeans were more likely than African Americans to have 12-month major depression. For African Americans, Black Caribbeans, and non-Latino whites, greater disability was consistently associated with higher rates of both 12-month MDD and lifetime MDD. This result is consistent with previous research documenting the strong relationships between disability and depression in older adults (Alexopoulos, 2005). This finding is particularly salient for African Americans and Black Caribbeans with MDD who generally have higher levels of impairment and are more persistently ill than their white counterparts, thus experiencing a greater burden of disease (Brown, Ahmed, Gary, & Milburn, 1995; Williams et al., 2007).

In an extensive analysis, Woodward and colleagues (2013) investigated psychiatric disorders and physical illness comorbidities associated with MDD. Due to sample size limitations, the sample for this analysis included adults aged 50 years and older. Roughly 45% of respondents with lifetime MDD also met criteria for 12-month MDD. Older whites and Caribbean blacks had significantly higher lifetime MDD prevalence than African Americans. White females and Black Caribbean males had the highest rates of lifetime MDD. Consistent with previous research, MDD was highly comorbid with other psychiatric and physical disorders. Among African Americans with 12-month MDD, 44.9% had a lifetime anxiety disorder, 29.6% had lifetime dysthymia, 27.4% had a lifetime substance disorder, and 25.5% reported lifetime suicidal ideation. There were also fairly high levels of comorbidity for 12-month MDD and physical health problems. The most common physical disorder among older African Americans with MDD was high blood pressure, followed by arthritis, heart/circulation, ulcers, asthma, and diabetes. Roughly half of all African Americans with a 12-month MDD rated their physical health as fair or poor (Table 1 summarizes findings from studies on psychiatric disorder among older African Americans).

**Table 1.** Summary of Selected Investigations of the Prevalence of Psychiatric Disorders and Use of Mental Health Services for Older Black Americans

Authors	*N*	Sample	Dependent variables	Major independent variables	Selected findings
Within Group Analysis of Psychiatric Disorders among Older African Americans					
Ford et al., 2007	837	NSAL, African Americans aged 55 and older	Any lifetime mood disorder Any lifetime anxiety disorder Any lifetime substance disorder Any lifetime disorder Any 12-month disorder	Demographics	One-quarter of sample reported one lifetime disorder and 20% had two or more. Nine percent had a 12-month disorder and 3% had two or more. Age, sex, education, and region were associated with the odds of having a lifetime disorder; demographic factors were unrelated to 12-month disorders
Chatters et al., 2008	837	NSAL, African Americans aged 55 and older	Any lifetime mood disorder Any lifetime anxiety disorder Any lifetime substance disorder Any lifetime disorder Number of lifetime mood, anxiety and substance disorders	Religious service attendance, nonorganizational religiosity, subjective religiosity	Controlling for health and disability, religious service attendance was inversely associated with having a mood disorder
Lincoln et al., 2010	837	NSAL, African Americans aged 55 and older	Any lifetime mood disorder Any lifetime anxiety disorder Number of lifetime mood and anxiety disorders	Emotional support with family Negative interactions with family	Negative interaction was associated with odds of having a lifetime mood disorder, a lifetime anxiety disorder and number of lifetime mood and anxiety disorders; emotional support unrelated to mood and anxiety disorders
Peterson et al., 2014	185	NSAL, African Americans aged 55 and older with at least one lifetime psychiatric disorder	Subjective well-being (happiness, life satisfaction)	12-month mood and anxiety disorder Lifetime psychiatric disorder Lifetime suicidal ideation Demographics	Lifetime suicidal ideation was associated with life satisfaction only; 12-month mood disorder negatively associated with both happiness and life satisfaction
Mouzon et al., 2017	837	NSAL, African Americans aged 55 and older	Any lifetime mood disorder Any lifetime anxiety disorder Any lifetime disorder Number of lifetime mood and anxiety disorders Depressive symptoms (CES-D) Psychological distress (K6)	Overall everyday discrimination Everyday racial discrimination Everyday nonracial discrimination	Everyday discrimination (whether racial, nonracial, or overall) is associated with higher risk of psychiatric disorders, depressive symptoms, and serious psychological distress
Kim et al., 2017	429	NSAL, African American and Black Caribbeans, aged 55 and older who indicated that their racial/ethnic background was main reason for their discriminatory experiences	Any 12-month disorder	Everyday racial discrimination	Racial discrimination related to higher odds of any past year psychiatric disorder; racial discrimination and disorder relationship strongest in West (compared to South)
Both Within and Between Group (Race/Ethnicity) Analysis of Psychiatric Disorders among Older Adults					
Jimenez et al., 2010	2,375	CPES, non-Latino whites, Asians, African Americans, Black Caribbeans, and Latinos, aged 60 and older	Any 12-month depressive disorder Any 12-month anxiety disorder Any 12-month substance disorder Any 12-month disorder Any lifetime depressive disorder Any lifetime anxiety disorder Any lifetime substance disorder Any lifetime disorder	Nativity	Older non-Latino whites exhibited a greater prevalence on several lifetime psychiatric disorders than older Asians, African-Americans, and Black Caribbeans.
Woodward et al., 2012	3,046	CPES, non-Latino whites, Asians, African Americans, Black Caribbeans, and Latinos aged 55 and older	Any lifetime mood disorder Any lifetime anxiety disorder	Demographics	Non-Latino whites and Latinos had higher prevalence rates of disorders, African Americans had lower prevalence of major depression and dysthymia
Kim et al., 2011	1,840	CPES, non-Latino whites, blacks, Latinos, and Asians, aged 60 and older	Any 12-month disorder Any 12-month anxiety disorder	SRMH Race, ethnicity	SRMH and white race associated with mood and anxiety; relationship between SRMH and psychiatric disorders strongest for non-Latino whites
Aranda et al., 2012	1,439	NSAL, African Americans, Black Caribbeans, and non-Latino whites, aged 55 and older	Lifetime MDD 12-month MDD	Demographics	Non-Latino whites had the highest odds of lifetime MDD Women had significantly greater odds of lifetime MDD compared with men.
Woodward et al., 2013	1,950	NSAL, African Americans, Black Caribbeans, non-Latino whites, aged 50 and older	Lifetime MDD 12-month MDD	Comorbid lifetime anxiety and substance disorders Comorbid physical health problems	MDD lifetime prevalence was 12.1%; 12-month rate was 5.2%. Older whites and Black Caribbeans had significantly higher lifetime prevalence than African Americans; 12-month rates were similar across the three groups. Black Caribbean men had higher rates of lifetime MDD
Joe et al., 2014	1,141	NSAL, African Americans, Black Caribbeans, aged 55 and older	Lifetime suicidal behavior (ideation and attempts)	14 DSM-IV disorders (mood, anxiety, substance use); Demographics	Lifetime prevalence of suicidal ideation (6.1%) and attempts (2.1%); being male, middle-aged, having poorer health, anxiety, and multiple DSM-IV disorders associated with higher risk of suicide attempts
Both Within and Between Group (Race/Ethnicity) Analysis of Mental Health Service Use for Psychiatric Disorders among Older Adults					
Neighbors et al., 2008	837	NSAL, African Americans, aged 55 or older	Psychiatric and nonpsychiatric mental health services, general medical care, and nonhealth care within past 12 months	Any 12-month mood, anxiety, or substance use disorders	Less than half with one disorder and half with two disorders used any service; mood disorder associated with higher service use
Kim et al., 2013	2,878	CPES, blacks and whites, aged 60 and older	Mental health service use	Region; demographic controls	Older blacks less likely to use mental health services overall. Black elders in the South were significantly less likely than whites to use mental health services; no racial differences in Northeast, West or Midwest regions.

*Note*: CES-D = Center for Epidemiologic Studies Depression scale; CPES = Collaborative Psychiatric Epidemiology Studies; DSM-IV = Diagnostic and Statistical Manual of Mental Disorders, Fourth Edition; K6 = Kessler Psychological Distress scale; MDD = major depressive disorder; NSAL = National Survey of American Life: Coping with Stress in the 21st Century; SRMH = self-rated mental health.

## Race and Gender Differences in Psychiatric Disorders Among Older Adults

Very few studies investigate gender differences in psychiatric disorders among African American older adults. This is partly due to the relatively small number of cases of older African Americans who have psychiatric disorders in national samples, including the NSAL. Ford and colleagues (2007) did not find a significant gender difference in the odds of having a lifetime mood disorder or lifetime anxiety disorder; however, men were more likely than women to have a lifetime substance disorder. Another analysis of adults aged 50 years and older (Woodward et al., 2013) found that Black Caribbean men had the highest rates of age-adjusted lifetime MDD in comparison to African American men and women and Black Caribbean women. Although the number of cases of older Black Caribbean men was small, this finding was consistent with previous work on adults aged 18 and older (Williams et al., 2007). As noted previously, in a study using a combined sample of older African Americans and Black Caribbeans, men were more likely than women to have had suicidal ideations (Joe et al., 2014).

## Depressive Symptoms

As compared to psychiatric disorders, considerably more research focuses on depressive symptoms, including studies on prevalence (Lincoln et al., 2010) and risk (Marshall-Fabien & Miller, 2016) and protective factors (Chatters et al., 2015). Given that African Americans have lower rates of psychiatric disorders than non-Latino whites, it would be expected that they also have fewer depressive symptoms. Although some work indicates that older African Americans have fewer depressive symptoms (Lincoln et al., 2010), most studies indicate that older African Americans have more depressive symptoms than their white counterparts (Barnes & Bates, 2017). This includes studies that measure depression by using a cutoff number for depressive symptoms (Barnes & Bates, 2017). At present, there are no adequate explanations for this counterintuitive pattern, including possible differential item functioning between blacks and whites in diagnostic interviews (Barnes & Bates, 2017). These documented paradoxes clearly warrant further study. However, a full discussion of research on depressive symptoms among older blacks is beyond the scope of this review.

## Use of Services for Psychiatric Disorders Among Older African Americans

Research on service use and treatment for psychiatric disorders of older African American using data from national probability samples is even more limited than research on psychiatric disorder prevalence rates. This is because of the very small sample sizes of older African Americans who have a psychiatric disorder, coupled with low levels of service utilization in this population. Consequently, some studies use the entire adult age range and then conduct analysis of age differences (Mackenzie et al., 2010). At best, these studies provide information about racial differences among adults, but fail to provide any direct information about service utilization and treatment experiences among older African Americans.

This section provides a brief overview of research on service use among adults across the life course, followed by research on older African Americans. First, it is important to acknowledge that, regardless of race, a large proportion of Americans do not receive any type of treatment for mental health problems. Within this group, African Americans are much less likely to receive services for depression, anxiety, and other mental health problems. An analysis of individuals with 12-month depressive disorder found that six out of 10 African Americans and four out of 10 non-Latino whites did not receive any past-year mental health treatment (Alegría et al., 2008). African Americans were less likely than whites to receive talk therapy or pharmacotherapy (Gonzalez et al., 2010; González et al., 2010a). In one of the relatively few studies of antidepressant use, Gonzalez and colleagues (2008) found that blacks (both African Americans and Black Caribbeans) had lower use than white adults. Furthermore, depression severity was positively associated with higher antidepressant use among whites but not among black adults. Non-Latino whites were also more likely than both African Americans and Black Caribbeans to use complementary and alternative medicines for psychiatric disorders (Woodward et al., 2009). An innovative analysis of informal and professional help-seeking for psychiatric disorders (Woodward et al., 2008) found that among black adults (both African Americans and Black Caribbeans), 41% used both professional services and informal support, 14% relied on professional services only, 23% used informal support only, and 22% did not receive any assistance.

Neighbors and colleagues (2007) found a curvilinear relationship between age and service use for 12-month psychiatric disorders. Both younger (18–29) and older (60+) African Americans were less likely to seek general medical services, psychiatrists, other mental health specialists (psychologists, social workers), and nonhealth services (religious advisors, counselors, social workers, group work seen in a nonhealth care setting) than were African Americans aged 30–44 and 45–59 years. In a subsequent analysis, Neighbors and colleagues (2008) investigated service use for 12-month psychiatric disorders among older African Americans in which roughly half of those with a disorder utilized some form of service. Older African Americans with any mood disorder were more likely to use services than persons with any anxiety or any substance disorder.

Another analysis of the CPES (Kim et al., 2013) using adults aged 60 and older found that regional differences helped explain the disparity in service use among older whites and African Americans. Older whites and African Americans had comparable levels of service utilization in the Northeast, Midwest or West. However, older African Americans in the South were significantly less likely to use mental health services. Collectively, these findings indicate that a significant proportion of African American older adults with mental health disorders do not receive professional help. This is a particularly significant issue in the South, where half of all older African Americans reside.

## Directions for Future Research

As this literature review indicates, information on the prevalence and correlates of psychiatric disorders among older African Americans is exceedingly scarce. Consequently, there is so much that we do not know, especially in comparison to what is known about psychiatric disorders among non-Latino whites. Further, we have no information concerning the significance of health issues such as obesity, binge eating, auditory impairments, vision impairments, and social isolation in relation to psychiatric disorders among older African Americans. Similarly, protective and risk factors important for the mental health status of older LGBT African Americans remain unexplored. In discussing future research directions, we will limit our discussion to four issues that are particularly important to the black population: (1) mass incarceration, (2) the growing population of older immigrants from Africa, (3) racial socialization and racial identity, and (4) racial diversity in the life course. We conclude this section by discussing methodological opportunities for advancing this research.

### Mass Incarceration

The family members of African Americans are disproportionately affected by mass incarceration. Lee, McCormick, Hicken, and Wildeman (2015) indicate that 44% of African American women and 34% of African American men have a family member who has been incarcerated, compared with 12% and 6% of white women and men, respectively. One in nine black children has an incarcerated parent (Wakefield & Wildeman, 2013). Additionally, the impact of mass incarceration extends well beyond the suspected “legal offender” (Comfort, 2007). Incarceration of a loved one exerts negative impacts on the health and well-being of the children, romantic partners, parents, and community members of prisoners and former prisoners (Lee et al., 2015). For instance, one study found that African Americans who had a family member who was currently incarcerated reported more psychological distress (Mouzon, Taylor, Nguyen, & Chatters, 2016). Similarly, Lee, Wildeman, Wang, Matusko, and Jackson (2014) found that among African American women, family member incarceration was associated with higher odds of obesity, heart attack, or stroke. Research on the impact of mass incarceration on distress and psychiatric disorders should focus on older African Americans who are parents and grandparents of incarcerated adult children, with specific attention to those who are grandparent caregivers of minor children whose parents are incarcerated (i.e., grandfamilies). The mental health impact of incarceration should be addressed in clinical situations with older African Americans, as well as at policy levels with regards to criminal justice reform and child and family welfare efforts.

### Foreign-Born Blacks and Immigration

Overall, the black foreign-born population in the United States is growing at a substantial rate from 816,000 in 1980 to 4.2 million in 2016. Since 2000, the black foreign-born population increased by 71% and in 2015 constituted 9% of the overall black population in the United States. Although migration from the Caribbean region has been steady, a sizable portion of black foreign-born persons in the United States are immigrants from African countries (e.g., Nigeria, Ethiopia, Ghana), which represents the fastest growing segment of this group. For example, the black African immigrant population numbered 570,000 in 2000, representing 24% of the overall black immigrant population. In 2013, black African immigrants numbered 1.4 million and in 2016 they represented 39% of the overall black immigrant population. In addition, foreign-born blacks from Nigeria, Kenya, and Ghana are distinctive with regard to educational level and are more likely to have a college degree than native-born African Americans and the overall U.S. population. These demographic trends indicate both increasing ethnic diversity within the overall black population, as well as in the black foreign-born groups that immigrate to the United States. In addition, prior findings of unexpectedly higher rates of depression and suicidal behaviors among Black Caribbean men (Joe et al., 2006) suggests that research focusing on black ethnic groups can provide a better understanding of potential differences in how sociodemographic and other correlates are associated with mental health. Future studies of foreign-born black immigrants from countries in Africa provide the opportunity to complement prior research on psychiatric disorders among older native-born African Americans, as well as black immigrants from the Caribbean region.

### Racial Identity and Racial Socialization

Another promising area of future research explores the impact of racial socialization and racial identity on psychiatric disorders. A large body of research on child and adolescent development indicates that racial socialization efforts with black children can instill a strong racial group identity that instills a high level of tolerance when confronted with instances of discrimination, unfair treatment, and insults based solely on race (Huguley, Wang, Vasquez, & Guo, 2019; Thornton, Chatters, Taylor, & Allen, 1990). Racial socialization is positively associated with self-esteem and well-being (Reynolds & Gonzales‐Backen, 2017), but the vast majority of this work is on adolescents. Future research should examine whether racial socialization or a strong racial identity are protective of psychiatric disorders across all age groups, including older African Americans. Researchers may also want to investigate whether interventions that instill a sense of pride and positive racial identity among African American children have spillover mental health effects for parents and grandparents.

### Racial Diversity in the Life Course

Life-course theory is a major cornerstone of gerontology and a powerful approach for examining how racial inequities and structural factors (e.g., poverty, inadequate health care, poor housing, environmental hazards) produce and maintain differences in exposures, trajectories, and the cumulative impacts of adverse social conditions that ultimately result in racial disparities in health (Gee, Hing, Mohammed, Tabor, & Williams, 2019; Jones et al., 2019; Walsemann, Gee, & Geronimus, 2009). Using a life-course perspective, Umberson and colleagues (2017) found that blacks are more likely than whites to experience deaths of family members at specific periods of the life course (from childhood through midlife: parents and sibling deaths; from young adulthood on: death of a child and spouse). These “off-time” deaths are especially powerful life events that have negative impacts on mental and physical health, as well as economic and educational trajectories for individuals (e.g., children, spouses) and family members (via “linked lives”). Innovative research on racial diversity in the life course should: (1) assess racial diversity in life-course events, their timing, and trajectories for individuals and families, (2) investigate both the cumulative and life stage-specific impacts on individuals, families, and communities, and (3) apply this knowledge to develop policies and practices that both eradicate social conditions that produce health disparities (i.e., “health in all policies”) and advance efforts to achieve health equity (Gee et al., 2019; Umberson et al., 2017). This holistic approach is essential in providing a more nuanced and thorough understanding of the various social conditions that are risk and protective factors for psychiatric disorders among older African Americans.

### Methodological Opportunities

The largest advances in understanding race and mental health have been made through the use of nationally representative survey data. The continued and expanded use of these important data sources are necessary for addressing acknowledged research gaps identified in this review. We also see considerable opportunities for adopting a data science framework to analyze data sources that have not been used in this line of research. For example, electronic health records (EHRs) consist of unstructured text data that have traditionally been examined using qualitative methods applied to a small set of EHRs. Recent advances in data science methods show promising results for extracting information from vast stores of unstructured text data. For example, Perron and colleagues (2019) used a data science methodology called machine learning methods to analyze a vast collection of unstructured text documents (>75,000) from electronic child welfare records. Their computer models extracted information on family substance abuse problems and domestic violence problems with an accuracy of more than 90% when compared to trained human coders. Human coders took over 20 min to review each document; the computer models performed the coding task on more than 75,000 records in less than 10 min.

The same methodology can be applied to the analysis of EHRs, with the potential to scale the analysis far beyond what is possible with traditional qualitative methods. Developing this area of research necessarily requires gaining access to available EHRs and thinking critically about what information can be reliably extracted. The most successful analyses will involve a mixed-methods approach. Qualitative methods can be used to understand what information can be extracted from these data, in addition to establishing reliable coding procedures for training computer models. Following this, a variety of off-the-shelf software applications can be used for implementing the quantitative analysis of the text data. Developing and applying a data science framework can maximize the usefulness of EHRs, provide a potentially rich source of information for examining mental health symptomology and service utilization, and greatly expand our understanding of mental health within older black populations in the United States.

## Conclusion

Research on the prevalence and correlates of psychiatric disorders among older Black Americans continues to make important contributions to understanding the etiology and risk and protective factors associated with these morbidities. This work enhances our understanding of current theoretical frameworks and models of the etiology of psychiatric disorders and the degree to which they align with the life trajectories, social experiences, and circumstances of older black adults. This comparison underscores significant gaps in knowledge and has prompted investigations of stressful life experiences, racial discrimination, and psychiatric disorders. This includes a nascent framework (Environmental Affordances Model) suggesting that individual coping behaviors may preserve mental well-being at the expense of physical health. Research exploring between-group (black/white) and within-group variability in sociodemographic correlates (gender, region), as well as ethnic (native-born African American/Black Caribbean) differences in psychiatric disorders has generated more nuanced profiles of the prevalence and risk and protective factors for specific groups of older black adults. Finally, research has increased our understanding of how social relationships and resources function as assets for mental health and well-being. Future research directions include addressing current gaps in our knowledge regarding the correlates and profiles for specific disorders (e.g., PTSD), assessing the impact of differential life events and trajectories (e.g., mass incarceration), examining population dynamics (e.g., immigration) and psychiatric disorders, and exploring coping strategies that draw upon family, friend, and religious resources among older Black Americans.

## Funding

The preparation of this manuscript was supported by a grant from the National Institute on Aging to R. J. Taylor (P30-AG015281).
